# Supplementary material for: Establishment and Its Utility of a Patient-Derived Cell Xenografts (PDCX) Model with Cryopreserved Cancer Cells from Human Tumor
Source: Cells. 2025 Feb 21;14(5):325. doi: 10.3390/cells14050325 (PMC11898490; doi:10.3390/cells14050325)
Supplement: Supplementary file 1 [file cells-14-00325-s001.zip › cells-3407587-supplementary.pdf]

Supplemental Table 1.

Table S1. Number of cancer cells obtained from 1 mg of M2 PDCX tumor

Cancer	Mouse ID	Dissociated Tumor (mg)	Counted total cell no. (x10^4)	Counted live cell no. (x10^4)	Cell Viability (%)	Total cell no. / 1mg (x10^4)	Live cell no. / 1mg (x10^4)
SC155 Cell	SC155C2M2-1R	280	2200	1758	79.7	7.86	6.28
	SC155C2M2-1L	1360	7620	6540	85.9	5.60	4.81
	SC155C2M2-2R	1440	7520	6320	84.3	5.22	4.39
	SC155C2M2-2L	730	5920	4760	80.1	8.11	6.52
Average (±SD)		952.5 (±475.8)	5815 (±2193.4)	4844.5 (±1909.6)	82.5 (±2.67)	6.70 (±1.30)	5.5 (±0.92)
SC156 Cell	SC156C1M2-1R	1060	6480	5720	88.6	6.11	5.40
	SC156C1M2-1L	610	4440	3820	86.0	7.28	6.26
	SC156C1M2-2R	890	5720	5080	88.8	6.43	5.71
	SC156C1M2-2L	1100	5920	4720	80.0	5.38	4.29
	SC156C3M2-1R	1010	7360	6360	86.1	7.29	6.30
	SC156C3M2-1L	630	4760	4120	86.2	7.56	6.54
	SC156C3M2-2R	520	3120	2660	85.2	6.00	5.12
	SC156C3M2-2L	480	2760	2520	90.9	5.75	5.25
Average (±SD)		787.5 (±238.3)	5070 (±1500.4)	4375 (±1280.7)	86.5 (±3.03)	6.48 (±0.76)	5.61 (±0.70)

Supplemental Table 2.

Table S2. Engraftment rates of PDCX_M1 according to the cryopreservation periods

Duration	Case	Cryopreserved Period	Engraftment	Engraftment rate
Short (< 100 days)	SC155	30	2/4	11/15 (73.3%)
	SC156	19	4/4	
	SC214	24	2/2	
	SC227	15	2/2	
	SC238	79	1/1	
	SC243	37	0/2	
Middle (100 ~ 365 days)	SC101	269	0/1	5/10 (50%)
	SC124	119	0/2	
	SC236	126	2/2	
	SC245	115	1/1	
	SC246	148	0/2	
	SC248	100	2/2	
Long (365 days <)	SC18	557	0/1	2/4 (50%)
	SC39	485	1/1	
	SC214	386	1/2	

Supplemental Figures 1. SGR comparisons between SC156 PDCX_M2 and SC156 PDTX_M2.

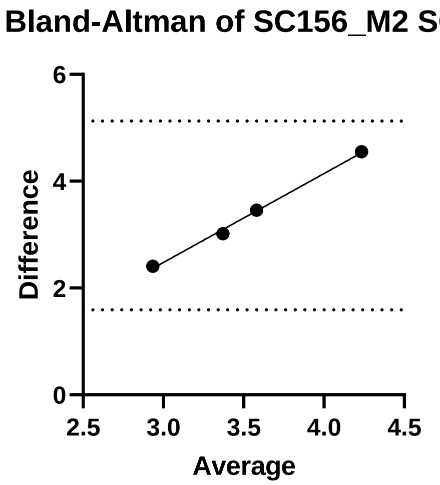

SC156 PDCX_M2		SC156 PDTX_M2		Average ((Method 1+Method 2)*0.5)	Difference (Method 2 – Method 1)
Tumor ID	SGR per 100 days (Method 1)	Tumor ID	SGR per 100 days (Method 2)		
SC156C1M2-1 Left	5.31	SC156T4M2-1 Left	1.85	3.580	3.46
SC156C1M2-2 Left	6.51	SC156T4M2-2 Left	1.96	4.235	4.55
SC156C2M2-1 Left	4.14	SC156T4M2-1 Right	1.73	2.935	2.41
SC156C3M2-1 Left	4.88	SC156T4M2-2 Right	1.86	3.370	3.02

Supplementary Figure 1. SGR comparisons between SC156 PDCX_M2 and SC156 PDTX_M2. The SGR values of SC156 PDCX_M2 were compared to that of SC156 PDTX_M2 by using Bland-Altman analysis in GraphPad Prism software. In this anlaysis, the difference is interpreted when the Difference (Values of Method 2 – Method 2) does not include 0, and the graph pattern reveals a positive or negative correlation with the Average (mean of SGR values). In this comparisons, the 95% limits of agreement ranged 1.591 to 5.129, and showed a significant positive linear pattern (R^2=0.9969).
